# Diurnal Variation in Trigeminal Pain Sensitivity in Mice

**DOI:** 10.3389/fnins.2021.703440

**Published:** 2021-08-02

**Authors:** Ayako Niiro, Sachi N. Ohno, Kanae A. Yamagata, Kazuaki Yamagata, Kazuo Tomita, Eriko Kuramoto, Yoshiaki Oda, Takahiro J. Nakamura, Wataru Nakamura, Mitsutaka Sugimura

**Affiliations:** ^1^Department of Dental Anesthesiology, Graduate School of Medical and Dental Sciences, Kagoshima University, Kagoshima, Japan; ^2^Department of Applied Pharmacology, Graduate School of Medical and Dental Sciences, Kagoshima University, Kagoshima, Japan; ^3^Department of Oral Anatomy and Cell Biology, Graduate School of Medical and Dental Sciences, Kagoshima University, Kagoshima, Japan; ^4^Unit of Basic Medical Sciences, Department of Oral Chrono-Physiology, Graduate School of Biomedical Sciences, Nagasaki University, Nagasaki, Japan; ^5^Laboratory of Animal Physiology, School of Agriculture, Meiji University, Kawasaki, Japan

**Keywords:** pain, day-night difference, formalin test, trigeminal nervous system, TRPA1

## Abstract

Management of time and circadian disruption is an extremely important factor in basic research on pain and analgesia. Although pain is known to vary throughout the day, the mechanism underlying this circadian variation remains largely unknown. In this study, we hypothesized that the process of pain transmission to the central nervous system (after receiving nociceptive stimuli from outside the body) would show day-night differences. Ten-week-old male mice were kept under a strict 12/12-h light/dark cycle for at least 10 days. Formalin was then injected into the second branch region of the trigeminal nerve and the duration of pain-related behaviors (PRBs) was assessed. Immunohistochemical staining was then performed, and the c-Fos-immunopositive cells in the trigeminal spinal tract subnucleus caudalis (Sp5C) were counted. The results showed that the duration of PRBs was longer and the number of c-Fos immunopositive cells in the Sp5C was higher at nighttime than during the day. In addition, the trigeminal ganglia (TG) were extracted from the mice and examined by quantitative real-time PCR to evaluate the daytime and nighttime expression of nociceptive receptors. The results showed that the mRNA expression of transient receptor potential ankyrin 1 in the TG was significantly higher at night than during the day. These results suggest that pain in the trigeminal nerve region is more intense at nighttime, when rodents are active, than during the daytime, partly due to differences in nociceptor expression.

## Introduction

In mammals, physiological functions and behaviors such as sleep, wakefulness, body temperature, and endocrine secretion are regulated by circadian rhythms to ensure that these functions are maximized throughout the day (Ruben et al., 2018; Rijo-Ferreira and Takahashi, 2019). In recent years, medical researchers have attempted to elucidate the causes of various diseases, including sleep disorders (Hirano et al., 2016; Patke et al., 2017), hypertension (Douma and Gumz, 2018), and cancer (Kettner et al., 2016; Sulli et al., 2018), in order to develop treatment strategies (Colwell, 2015) by applying their knowledge regarding biological clock mechanisms. As for pain research, chronobiological studies have revealed the circadian variability of pain. However, these studies have mainly focused on behavioral findings when animals are subjected to nociceptive stimuli (Rosenfeld and Rice, 1979; Pickard, 1987; Konecka and Sroczynska, 1998), and only a few studies have attempted to elucidate the related molecular mechanisms (Koyanagi et al., 2016). In addition, despite numerous basic research studies on pain and their applications to clinical practice, many studies did not take into account the fact that nociception changes throughout the day (Baliki et al., 2010; Christianson et al., 2010). The concept of time is one of the most important factors in basic research on pain and analgesia, and the identification of the circadian variation of pain and underlying molecular mechanisms is expected to lead to more effective methods of pain control.

The orofacial region is the area of the body with the highest concentration of nerves. It is also the area that experiences the most severe acute clinical pain (Sessle, 2000). In the orofacial region, nociceptive stimuli are received by the trigeminal ganglion (TG) neurons (i.e., primary nociceptive neurons), and are subsequently transmitted to secondary neurons in the trigeminal spinal tract subnucleus caudalis (Sp5C). Nociceptive information is transmitted further upstream *via* the thalamus to the somatosensory cortex, where this information is perceived as pain (Van der Cruyssen and Politis, 2018). We sought to elucidate whether this pathway of pain transmission from the peripheral nerves to the central nervous system included elements responsible for day-night differences in pain perception. We focused on the expression of transducers located at the peripheral edges of primary nociceptive neurons, which generate action potential responses to various nociceptive stimuli. Among them, transient receptor potential ankyrin 1 (TRPA1) is known to be activated not only by various exogenous stimuli, but also by endogenous substances involved in inflammation (Trevisani et al., 2007; Nilius et al., 2012; Dai, 2016). TRPA1 is widely expressed in the sensory nervous system, including the TG (Kobayashi et al., 2005). Since TRPA1 is at the forefront of nociception, we hypothesized that this would be a key factor mediating behavioral-level day-night differences in pain. To test this hypothesis, we reproduced a mouse model of acute, persistent pain, in which the second branch region of the trigeminal nerve was injected with formalin (Luccarini et al., 2006; Bornhof et al., 2011). We then examined day-night differences in nociception at the behavioral and cellular levels. We also discussed the relationship between TRPA1 expression in the trigeminal nerve and day-night differences in nociception.

## Materials and Methods

### Ethics Statement

All procedures involving animals were performed in accordance with the National Institutes of Health Guide for the Care and Use of Laboratory Animals. This study was approved by the Animal Care and Use Committee of Kagoshima University (permission #D17008, D19036).

### Animals

Ten-week-old male C57BL/6J mice were purchased from Japan SLC, Inc. (Shizuoka, Japan) and housed in a temperature-controlled (24–25°C) quiet room under a 12/12-h light/dark cycle [i.e., light on was defined as Zeitgeber time (ZT) 0, light off was defined as ZT12] for at least 10 days, with food and water available *ad libitum*. Thirty-two mice were used for the formalin test and the immunohistochemistry experiments (saline-daytime; *n* = 8, saline-nighttime; *n* = 8, formalin-daytime; *n* = 8, formalin-nighttime, *n* = 8), and 18 mice were used for quantitative RT-PCR (qPCR) experiments (daytime; *n* = 9, nighttime; *n* = 9). Although the sample size was not pre-determined using statistical methods, the sample size for this study was set with reference to previous studies in which the formalin test was performed in the facial region of mice (Luccarini et al., 2006; Zhang et al., 2018).

### Formalin Test

Mice were placed individually in custom-made experimental cages (315 mm × 185 mm × 245 mm), with mirrors on three sides of a clear plastic box. Formalin was prepared from stock formalin (an aqueous solution of 37% formaldehyde; FUJIFILM Wako Chemicals, Osaka, Japan) and diluted to 5% in saline. After a 30-min habituation, either formalin (5%, 10 μL) or saline (10 μL) was injected subcutaneously into the right upper lip using a 26-gauge needle attached to a Hamilton (701, Hamilton Company, Reno, NV, United States) syringe. After the injection, the mouse was immediately returned to the experimental cage and recorded for 45 min using an infrared camera (HX-A1H; Panasonic, Tokyo, Japan). Because the camera was capable of capturing images in the dark field, we performed recordings under infrared light using C-Light (Sun Mechatronics, Tokyo, Japan), which did not affect the mice at night. Rubbing the injection site with the forepaw was defined as pain-related behavior (PRB), and the duration of time for which animals exhibited this behavior was measured cumulatively. The recording time of 45 min was divided into 15 blocks of 3 min each, and we distinguished two phases following the formalin injection [phase I (0–3 min) and phase II (15–39 min)], in accordance with a previous report (Luccarini et al., 2006). The duration of PRB was assessed by an experimenter who was blinded to the treatment condition. The experiments were conducted at two time points: at daytime (ZT6) and nighttime (ZT18).

### Immunohistochemistry

After the formalin test (2 h after formalin injection, ZT8 or ZT20), the mice were anesthetized by an intraperitoneal injection of sodium pentobarbital (60 mg/kg) and perfused intracardially with 10 mL of 5 mM sodium phosphate (pH 7.4)-buffered 0.9% saline (PBS), followed by 50 mL of 4% formaldehyde, 75%-saturated picric acid, and 0.1 M Na_2_HPO_4_ (adjusted with NaOH to pH 7.0). The brains were removed and post-fixed for 3 h at 4°C with the same fixative. After cryoprotection with 30% sucrose in PBS, the caudal brainstems were cut into 40-μm-thick serial coronal sections on a freezing microtome, and the sections were collected serially in PBS. Every third section was processed for further immunohistochemical analysis and all subsequent incubations were performed at room temperature (24–26°C). The sections were incubated overnight with 1 μg/mL rabbit antibody to c-Fos (ABE457; Sigma-Aldrich, St Louis, MO, United States) or 1 μg/mL rabbit antibody to calcitonin gene-related peptide (CGRP) (C8198; Sigma) in PBS containing 0.3% Triton X-100 (PBS-X), 0.12% lambda-carrageenan, 0.02% sodium azide, and 1% donkey serum (AB237258; Jackson Immuno Research Laboratories, Inc., Bar Harbor, ME, United States) (PBS-XCD). After rinsing with PBS-X, the sections were incubated for 1 h with 10 μg/mL biotinylated anti-rabbit IgG donkey antibody (AP182P; Merck Millipore, Burlington, MA, United States) and then for 1 h with avidin-biotinylated peroxidase complex (ABC) (1:100; Elite variety, Vector, Burlingame, CA, United States) in PBS-X. After rinsing in PBS, the bound peroxidase was developed into a brown precipitate by reaction for 10–20 min with 0.02% diaminobenzidine-4HCl (DAB; D5637-1G, Sigma) and 0.003% H_2_O_2_ in 50-mM Tris-HCl (pH 7.6). All stained sections were serially mounted onto glass slides, dried, dehydrated in an ethanol series, cleared in xylene, and finally mounted with a coverslip. Sections adjacent to sections stained for c-Fos were counterstained for Nissl with 0.2% cresyl violet to identify the cytoarchitecture. The cytoarchitecture of the brainstem, including the Sp5C, was mainly determined according to the atlas of Paxinos and Franklin (2012). Laminae boundaries were determined with reference to previous studies using mice (Romero-Reyes et al., 2013) and in CGRP-immunostained sections with adequate reference to the study by Sugimoto et al. (1997), since CGRP-immunoreactivity is considered to be selectively located in laminae I/II of the Sp5C.

Sections were automatically captured in a large color image through the normal mode of a TOCO digital slide scanner (CLARO, Aomori, Japan) with a 10 × objective lens (Zeiss, Oberkochen, Germany). In the normal mode, the scanner obtained 11 images with differently focused images at each site, selected the best-focused image at each site, and fused the partially overlapping images into a large image (Ohno et al., 2012). For each image, the average number of c-Fos positive cells in the superficial layer (laminae I/II) of Sp5C (8.12–8.24 mm posterior to bregma) ipsilateral to the injection side was counted manually using CANVAS X software (ACD Systems International Inc., Victoria, BC, Canada). The c-Fos positive cells in each section were checked under a microscope equipped with a 20 × objective lens (SPlanApo20; numerical aperture = 0.7; Nikon Tokyo, Japan), while frequently changing the microscopic focus. At least three sections per animal were analyzed, and the average numbers of c-Fos-positive cells during daytime and nighttime with and without formalin were compared.

### qPCR

The mice of this group were housed under the same conditions as those used in the formalin test. After euthanizing the mice by cervical dislocation, the TG on both sides was immediately removed at ZT8 (daytime) and ZT20 (nighttime). Total RNA was extracted using ISOGEN (Nippon Gene, Toyama, Japan), as recommended by the manufacturer. One microgram of total RNA was reverse-transcribed to cDNA by using oligo-dT adapter primers and ReverTra Ace (TOYOBO, Osaka, Japan). A cDNA equivalent of 2 ng of total RNA was used for qPCR. The qPCR reactions were performed with an ABI 7300 real-time PCR system (Applied Biosystems, Foster City, CA, United States) using THUNDERBIRD^®^ qPCR Mix (TOYOBO, Osaka, Japan). β-actin was used as the loading control. cDNA was amplified as follows: one cycle at 95°C for 10 min, followed by 40 cycles at 95°C for 10 s, 55°C for 10 s, and 72°C for 30 s. Each experiment was performed in triplicate. The primer sequences used in this experiment are provided below (Sasaguri et al., 2018; Tomita et al., 2019).

TRPA1 F: 5′-GTCCAGGGCGTTGTCT-3′
TRPA1 R: 5′-CGTGATGCAGAGGACAGAGAT-3′
β− actin F: 5′-AGAGCTACGAGCTGCCTGAC-3′
β− actin R: 5′-AGCACTGTGTTGGCGTACAG-3′

### Statistical Analysis

All results are presented as means ± standard errors of the means (SEM), and differences were considered statistically significant at *P* < 0.05. The two-way analysis of variance (ANOVA), *post hoc* Scheffé’s *F* tests, and unpaired Student’s *t*-tests were used for comparative analyses.

## Results

### Temporal Differences in the PRBs of Mice

**FIGURE 1:**
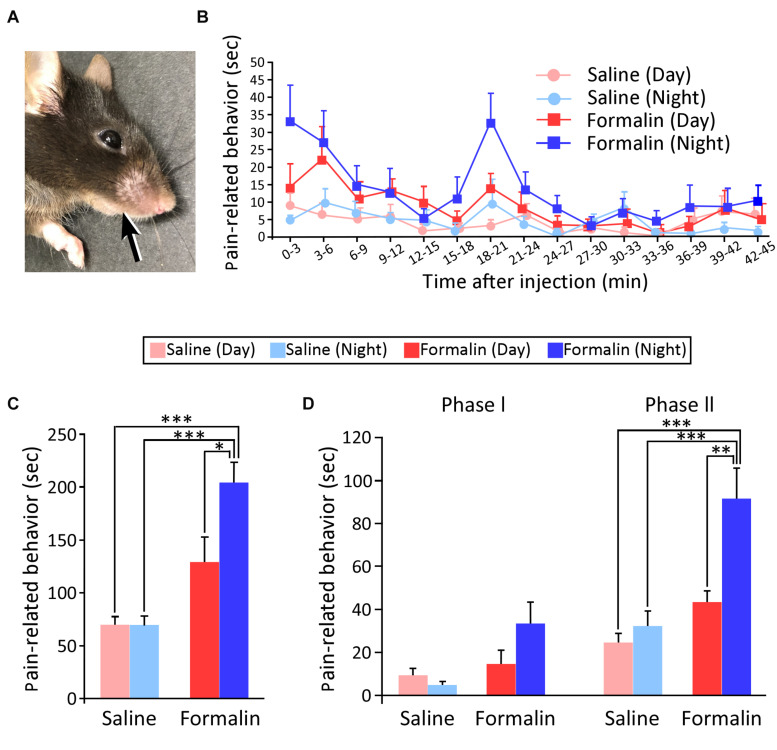
Injection site of the solution and temporal differences in pain-related behaviors (PRBs) in mice. Injection site of the solution **(A)**. Time courses of PRBs observed after the injection of either saline or formalin into the upper lip at daytime and nighttime. The mean number of seconds that mice spent rubbing is plotted for each 3-min bin over the 45-min post-injection observation period **(B)**. Total duration of PRBs observed at 45 min after formalin injection. Statistical analysis was performed using two-way ANOVA followed by Scheffé’s *F*-test. ^∗^*P* < 0.05 and ^∗∗∗^*P* < 0.001, respectively, **(C)**. Subtotal durations of PRB during phases I (0–3 min) and II (15–39 min) (Statistical analysis was performed using two-way ANOVA followed by Scheffé’s *F*-test. ^∗∗^*P* < 0.01 and ^∗∗∗^*P* < 0.001, respectively) **(D)**. Sample size, *n* = 8 in each group. Each value presents the mean ± standard error of the mean.

First, we reproduced a model of acute, persistent pain by injecting formalin into the right upper lip of mice (Figure 1A) and examined whether PRBs showed day-night differences at the behavioral level. Figure 1B shows the time course of the PRBs in the daytime and nighttime observed after the injection of saline or formalin into the right upper lip (saline-daytime; *n* = 8, saline-nighttime; *n* = 8, formalin-daytime; *n* = 8, formalin-nighttime, *n* = 8). The mean duration of the PRBs was plotted for each 3-min bin over the 45-min post-injection observation period. The PRB duration in the formalin group appeared to be longer in the nighttime than in the daytime, but there was no statistically significant difference in any 3-min bin. At nighttime, the formalin group showed a characteristic bimodal pattern with two peaks. The first peak emerged immediately after the administration of formalin, and the second one appeared at approximately 18–21 min.

Figure 1C shows the total duration of PRBs. First, for the two-way ANOVA, we defined the factor as the time of the experiment (daytime or nighttime) and the condition as the type of solution injected (saline or formalin). Next, we examined whether these two affect each other, and found that there was the interaction (*F*_1_,_28_ = 5.152, *P* = 0.031; two-way ANOVA). Accordingly, we conducted a multiple comparison test for all groups. The saline group did not show a significant difference in the duration of PRBs between the daytime and nighttime. Next, on comparing injections of 5% formalin and saline, there was no significant increase in the duration of PRBs in the daytime (saline-daytime; 69.4 ± 8.0 s, formalin-daytime; 128.6 ± 24.0 s), but there was a significant increase in the duration of PRBs in the nighttime (saline-nighttime; 69.1 ± 8.9 s, formalin-nighttime; 203.6 ± 19.4 s, *P* < 0.001, Scheffé’s *F* test). Interestingly, in the formalin group, the duration of PRBs was found to be significantly longer in the nighttime than in the daytime (*P* = 0.031, Scheffé’s *F* test). These results indicate that the pain sensitivity was higher at nighttime than at daytime when assessed at the animal behavioral level.

Since previous studies have suggested that phase I primarily reflects the pain response due to the direct stimulation of nerve endings by formalin and phase II reflects a secondary inflammatory pain response (Shibata et al., 1989), the duration of PRBs was divided into phase I (0–3 min) and phase II (15–39 min) (Figure 1D). In this figure, phase I and phase II represent a total PRB duration of 3 min and 24 min, respectively. In phase I, two-way ANOVA was performed for two factors, namely, the solution injected (saline or formalin) and the time of the experiment (daytime or nighttime); there was no significant difference for time of the experiment (*F*_1_,_28_ = 1.299, *P* = 0.264), but there was a significant difference for the solution injected (*F*_1_,_28_ = 7.109, *P* = 0.013), with no interaction (*F*_1_,_28_ = 3.436, *P* = 0.074). Thus, in phase I, there was a significant variation depending on the solution injected. In phase II, as in phase I, we first conducted a two-way ANOVA and found that there was the interaction between the solution injected and the time of the experiment (*F*_1_,_28_ = 5.365, *P* = 0.028). Next, a multiple comparison test showed that there was no significant difference in the saline group (daytime; 24.6 ± 4.4 s, nighttime; 32.3 ± 7.2 s). In the formalin group, PRB duration was of longer at nighttime than at daytime (daytime; 43.5 ± 5.3 s, nighttime; 91.5 ± 14.3 s, Scheffé’s *F* test, *P* = 0.006). These results indicate that the inflammatory pain response was greater during the nighttime than during the daytime.

### Temporal Differences in the Number of c-Fos-Positive Cells in the Sp5C

**FIGURE 2:**
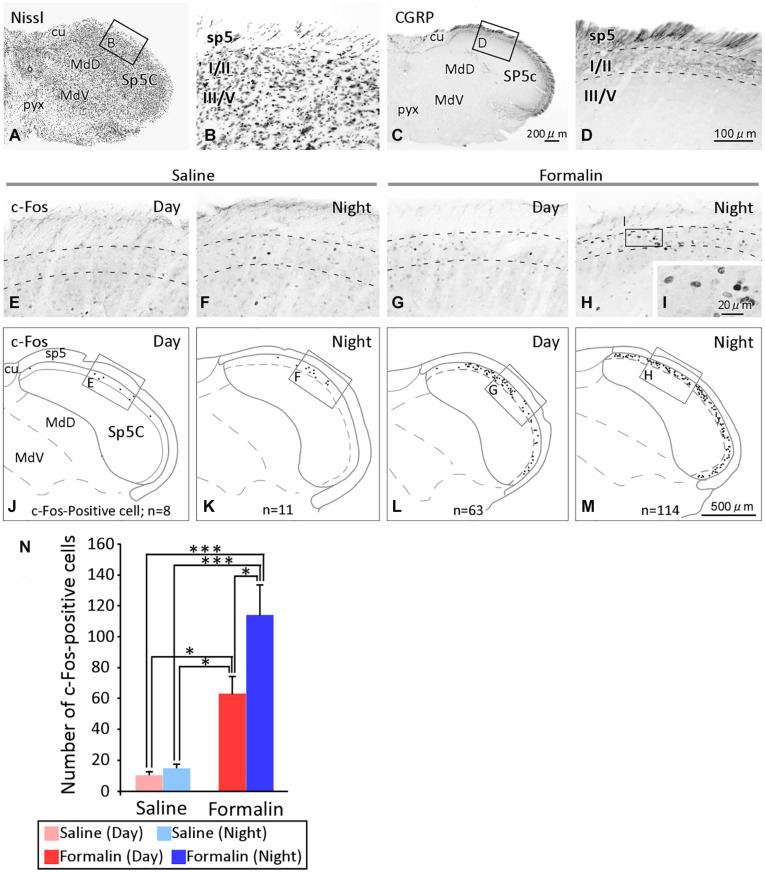
Temporal differences in the c-Fos expression in the spinal tract subnucleus caudalis (Sp5C). The cytoarchitecture and lamina structure of the Sp5C **(A–D)**. Calcitonin gene-related peptide (CGRP) immunoreactivity was observed selectively in laminae I/II, but not laminae III/IV, of the Sp5C **(C,D)**. c-Fos immunoreactivity in laminae I/II of the Sp5C ipsilateral to the saline injection site [**(E)** daytime; **(F)** nighttime]or formalin site [**(G)** daytime; **(H)** nighttime)] injection. The representative distribution of c-Fos positive cells in laminae I/II of the Sp5C **(J–M)**. The c-Fos-positive cells in each section were plotted onto a coronal plane under a microscope with a 20 × objective lens (SPlanApo20; numerical aperture = 0.7; Nikon Tokyo, Japan), while frequently changing the microscopic focus. Images of c-Fos positive cells at high magnification with a 40 × objective lens (UPlanSApo40; numerical aperture = 0.9; Olympus Tokyo, Japan) **(I)**. The number of c-Fos immunoreactive cells in laminae I/II of the Sp5C [**(N)** mean ± standard error of the mean, ^∗^*P* < 0.05, ^∗∗∗^*P* < 0.001, Scheffé’s *F*-test). In the formalin group, the number of c-fos-expressing cells was higher at nighttime than at daytime. cu, cuneate fasciculus; MdD, medullary reticular nucleus, dorsal part; MdV, medullary reticular nucleus, ventral part; pyx, pyramidal decussation; sp5, spinal trigeminal tract; for the other abbreviations, see the text. The scale bar in panel **(C)** applies to panels **(A,C)**, that in panel **(D)** applies to panels **(B,D–H)**, and that in panel **(M)** applies to panels **(J–M)**.

We subsequently examined how nociceptive stimuli received by peripheral nerves were transmitted to secondary neurons in the central nervous system. We used c-Fos as a marker of nociception in the present study because c-Fos has previously been shown to be an excellent marker for examining neurons activated by various nociceptive stimuli (Hunt et al., 1987; Bullitt, 1991; Nomura et al., 2002). In previous studies in rats, c-Fos expression was overwhelmingly observed on the ipsilateral side in comparison with the contralateral side (Veres et al., 2017), and it was observed in the rostrocaudal direction throughout the Sp5C (Zhou et al., 1999). Furthermore, it has been reported in studies using mice that the expression of c-Fos-positive cells is mainly found in laminae I and II, which receive nociceptive-specific input, as well as in the spinal dorsal horn (Romero-Reyes et al., 2013). Therefore, in this study, we counted the number of c-Fos positive cells located in laminae I/II (Figures 2A–D) of the Sp5C on the ipsilateral side of the formalin injection and found a difference, as shown in Figures 2E–L. The results for c-Fos expression were obtained from eight animals in each group that underwent the formalin test without any deficiency. The numbers of c-Fos-positive cells in laminae I/II of the Sp5C per section in the daytime and nighttime were 10.3 ± 2.5 and 14.5 ± 2.9 after the injection of saline, and 63.0 ± 11.1 and 114.5 ± 19.1 after the injection of formalin, respectively, (Figure 2N). We also conducted a two-way ANOVA and found that there was the interaction between the solution injected and the time of the experiment (*F*_1_,_28_ = 4.435, *P* = 0.044). The formalin groups showed statistically significantly higher numbers of c-Fos-positive cells compared to those in the saline groups, both during the daytime and during the nighttime (daytime, *P* = 0.023 and nighttime, *P* < 0.001, Scheffé’s *F* test). Furthermore, the number of c-Fos-positive cells did not significantly differ between daytime and nighttime in the saline groups, but was significantly higher during the nighttime than during the daytime in the formalin groups (*P* = 0.028, Scheffé’s *F* test).

### Temporal Differences in the mRNA Expression of TRPA1 in the TG

**FIGURE 3:**
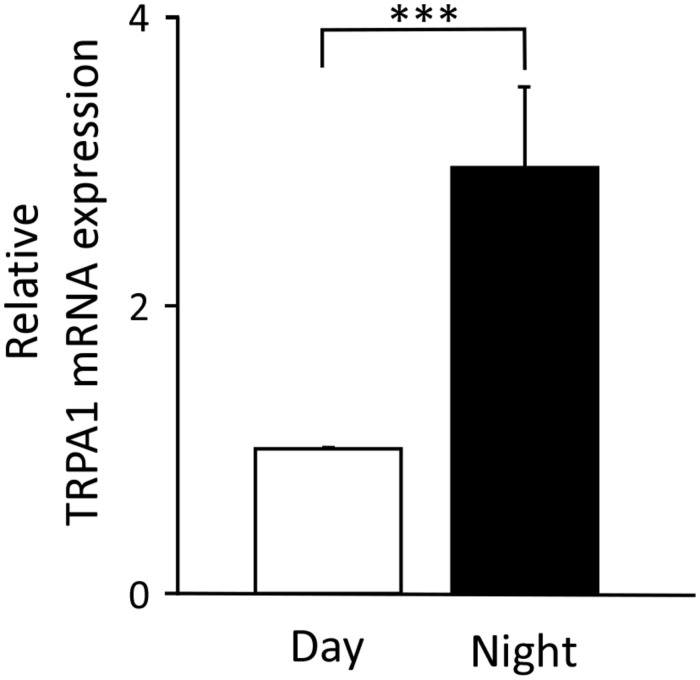
Temporal differences in the levels of transient receptor potential ankyrin 1 (TRPA1) mRNA expression in trigeminal ganglia. The relative value at nighttime when the daytime mRNA expression was set to 1 (mean ± standard error of the mean, ^∗∗∗^*P* < 0.001, Student’s *t*-test).

We searched for the causes of the day-night differences in nociception in the Sp5C. Because the stimuli applied to the upper lip were the same in both the daytime and the nighttime, we hypothesized that there would be day-night differences in the expression of transducers that convert nociceptive stimuli into electrical activity. Therefore, we performed qPCR to investigate gene expression changes in the TG, where the cell bodies of nociceptive neurons are located. We targeted TRPA1, which is reported to be receptive to a variety of chemical stimuli, including formalin. Figure 3 shows that the mRNA expression levels of TRPA1 in the TG were significantly higher at nighttime than during the daytime (*P* < 0.001, unpaired Student’s *t*-test).

## Discussion

In the present study, we hypothesized that the reception of nociceptive stimuli in the peripheral tissues of the neural pathways through which pain is transmitted would show day-night differences, and that this would be the key factor influencing behavioral-level day-night differences in pain. Consistent with this hypothesis, both the baseline expression of TRPA1 mRNA in the TG, which was higher at nighttime, and the higher number of c-Fos-positive cells in the Sp5C, induced by noxious formalin stimulation at nighttime, suggest that pain sensitivity in mice is higher at nighttime, when the animals are active.

More than 40 years have passed since the plantar formalin test was first reported by Dubuisson and Dennis (1977) and during this time this method has been established as a reliable means of assessing the nociceptive response in mice and rats (Tjølsen et al., 1992; Saddi and Abbott, 2000). For the assessment of orofacial pain, Luccarini et al. (2006) adapted the formalin test in rats and mice to assess nociceptive processes in the orofacial region (Clavelou et al., 1995; Luccarini et al., 2004, 2006). Therefore, we believe that the formalin test used in this study is suitable for assessing acute pain in the orofacial region. The present study is the first to address day-night differences in nociception in a trigeminal-innervated region, which is the site of densest innervation and frequent clinical acute pain (Figure 1). Pain response has been reported to show a characteristic biphasic pattern in formalin-based models of inflammatory pain. This has been confirmed by differences in pain response under various analgesic drugs. Specifically, central narcotic analgesics, such as morphine, suppress phases I and II, whereas anti-inflammatory analgesics, such as aspirin, which mainly act on the periphery, are known to suppress only phase II and almost never suppress phase I (Shibata et al., 1989). In the present results, the phase-I pain response to formalin administration (i.e., the direct pain response to formalin stimulation) did not significantly differ between daytime and nighttime. However, in phase II (formalin-induced inflammatory pain), the PRB duration was significantly longer during the nighttime than during the daytime. These results suggest that more anti-inflammatory analgesics are needed at night for pain caused by the inflammatory response.

Previous studies have shown that the immediate-early gene, c-Fos mRNA, is activated within minutes after acute nociception (Draisci and Iadarola, 1989), whereas c-Fos protein expression begins at 30 min after stimulation peaks at 1–2 h (Hunt et al., 1987; Presley et al., 1990; Williams et al., 1990). Therefore, the expression of c-Fos in Sp5C obtained in this study is considered to be the result of a combination of both acute pain caused by formalin stimulation (phase I) and pain caused by inflammation (phase II). Furthermore, as the formalin test in the present study was performed without anesthesia, it is possible that the expression of c-Fos includes all the procedures such as insertion of the needle, injection of the solution, and scraping of the injection site, compared to that in studies that observed pure neural responses to nociception under anesthesia (Kubota et al., 2007). However, c-Fos expression was more extended during the nighttime than during the daytime in the formalin group, suggesting that the transmission of invasion from primary to secondary neurons by this series of procedures was greater at nighttime, and that mice are more receptive to pain at nighttime than during the daytime (Figure 2).

**FIGURE 4:**
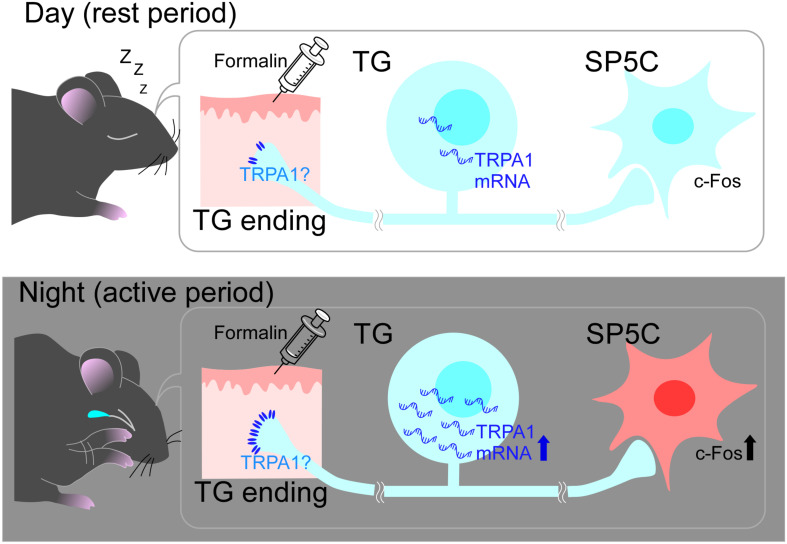
Schematic diagram of the mechanism of diurnal variation in pain sensitivity. During the nighttime, trigeminal ganglia (TG) might express transient receptor potential ankyrin 1. (TRPA1) to a greater extent than that during the daytime and activate the pain-sensation pathway more intensively. This would cause more vigorous and long-lasting pain-related behaviors (PRBs) in mice at nighttime than at daytime. See text for more details.

Transient receptor potential ankyrin 1 is a polymodal cation channel, which was reported in 2003 as a channel activated by nociceptive cold stimuli (Story et al., 2003). Anatomically, TRPA1 is predominantly found in C- and A-delta fibers, which are nociceptors in the trigeminal nerve, and is largely co-expressed with transient receptor potential cation channel subfamily V member 1 (TRPV1) (Caterina et al., 1997). In contrast, it is not co-expressed with transient receptor potential cation channel subfamily M member 8 (TRPM8), which is predominantly found in non-nociceptive fibers (Kobayashi et al., 2005). This is one of the reasons why TRPA1 has been studied as a pain-related channel (Dai, 2016). TRPA1 is not only activated by a variety of exogenous substances, including formalin (Dai, 2016), but also by endogenous substances involved in inflammation (Nilius et al., 2012). As formalin was used as a stimulant in the present study, we investigated whether the expression of TRPA1 showed a day-night difference. The results showed that TRPA1 mRNA expression was significantly higher during the nighttime than during the daytime (Figure 3), suggesting that formalin stimuli converted into electrical signals *via* TRPA1 are higher at nighttime than at daytime. Thus, when the same level of stimulation is applied to the trigeminal nerve during the daytime and nighttime, the amount of information transmitted as nociceptive stimuli will be higher at nighttime because TRPA1 is expressed more to a greater extent during the nighttime in the default state. Furthermore, TRPA1 is not only activated by endogenous substances, especially those involved in inflammation, but it is also known to trigger the inflammatory response itself (Nilius et al., 2012). Phase II of the formalin study reflected an inflammatory pain response and the results of the present behavioral experiment showed that PRBs lasted significantly longer in the nighttime than in the daytime in Phase II, which may be partly due to the stronger progression of the TRPA1-induced inflammatory response. These results suggest that differences in TRPA1 expression are transmitted to neural pathways beyond secondary neurons and manifest as a day-night difference at the behavioral level (Figure 4).

Pain undergoes various modifications after nociceptive stimuli are applied to peripheral tissues and is expressed as PRB. Indeed, in the present study, we did not consider mechanisms of pain suppression, including the descending inhibitory system; thus, it is possible that we only captured one aspect of pain transmission. As mentioned earlier, phase II of formalin-stimulated PRBs evaluates inflammatory pain, whereas the inflammatory response itself is thought to result in the release of endogenous opioids (Shibata et al., 1989). It is possible that a higher number of endogenous opioids were released during the daytime than during the nighttime, reactively suppressing pain during the daytime and relatively increasing sensitivity to pain during the nighttime. However, the results obtained in this study showed that mice raised in a strict light-dark cycle showed a distinct difference in pain sensitivity between daytime and nighttime when observed at the behavioral level, although the inhibitory system was not examined. The results of the present study demonstrate the importance of the strict control of time in future pain research. This is because it has been shown that exposure to light during the nighttime, even for a short duration, greatly disturbs the biological clock of laboratory animals (Morin, 2015), and how pain changes when the biological clock is disrupted is one of our interests not only in basic research but also in clinical practice. Mammalian cells are clocked 24 h a day by a transcription-translation feedback loop consisting of dozens of clock genes. In *Drosophila*, TRPA1 has been shown to be involved in temperature-dependent behavioral rhythms and diapause (Roessingh et al., 2015; Das et al., 2016). These reports suggest that TRPA1 is related to clock genes and is closely related to circadian rhythms in mammals, but further studies are needed to elucidate the relevant molecular mechanisms. In the future, we plan to conduct further studies, such as an assessment of day–night differences in pain, by using clock gene-knockout mice, TRPA1-knockout mice, and additional methodology.

In conclusion, in the present study, mice raised in a strict light-dark cycle were more sensitive to pain during the nighttime than during the daytime, and the results suggest that the day–night difference in the expression of the nociceptor TRPA1 may be responsible for this phenomenon (Figure 4).

## Data Availability Statement

The raw data supporting the conclusion of this article will be made available by the authors, without undue reservation.

## Ethics Statement

The animal study was reviewed and approved by the Animal Care and Use Committee of Kagoshima University.

## Author Contributions

AN contributed in conception and design of the study, performed all the experiments, prepared the figures, and writing the manuscript under the advice of SO and MS. SO designed the study with AN, performed the experiments, interpreted the results of experiments, and revised the manuscript. KAY supported the analysis of the formalin test and the maintenance of the mice. KY provided advice on how to conduct and interpret research on pain. KT provided technical support on real-time PCR. EK provided technical support for immunostaining and the preparation of Figure 2. WN, TN, and YO gave advice on how to maintain mice and how to conduct experiments at night from the point of view of chronobiology. All authors contributed to manuscript revision, read, and approved the submitted version.

## Conflict of Interest

The authors declare that the research was conducted in the absence of any commercial or financial relationships that could be construed as a potential conflict of interest.

## Publisher’s Note

All claims expressed in this article are solely those of the authors and do not necessarily represent those of their affiliated organizations, or those of the publisher, the editors and the reviewers. Any product that may be evaluated in this article, or claim that may be made by its manufacturer, is not guaranteed or endorsed by the publisher.
